# Mechanistic Model for Cancer Growth and Response to Chemotherapy

**DOI:** 10.1155/2017/3676295

**Published:** 2017-08-27

**Authors:** Eman Simbawa

**Affiliations:** Department of Mathematics, Faculty of Science, King Abdulaziz University, Jeddah 21589, Saudi Arabia

## Abstract

Cancer treatment has developed over the years; however not all patients respond to this treatment, and therefore further research is needed. In this paper, we employ mathematical modeling to understand the behavior of cancer and its interaction with therapy. We study a drug delivery and drug-cell interaction model along with cell proliferation. Due to the fact that cancer cells grow when there are enough nutrients and oxygen, proliferation can be a barrier against a response to therapy. To understand the effect of this factor, we perform numerical simulations of the model for different values of the parameters with a continuous delivery of the drug. The numerical results showed that cancer dies after short apoptotic cycles if the cancer is highly vascularized or if the penetration of the drug is high. This suggests promoting angiogenesis or perfusion of the drug. This result is similar to the situation where proliferation is not considered since the constant release of drug overcomes the growth of the cells and thus the effect of proliferation can be neglected.

## 1. Introduction

There have been extensive studies regarding cancer as it is one of the leading causes of death [1]. The main goal of these studies is to find the most effective therapy with minimal patient suffering. One aspect of research includes mathematical modeling which offers a platform to study cancer without losing patients' lives [2–6]. It provides an insightful tool to explore and predict the growth of cancer as well as the response to therapy by using biological and physical properties. These models are then validated using in vivo and in vitro experiments as well as patients' data. The results help oncologists customize therapy for each patient by understanding the physical and biological barriers that make some cancer patients not respond to therapy.

In light of cell population, one could use ordinary differential equations (ODEs) to describe the evolution of the total number of cancer cells with and without chemotherapy [7]; however, since cancer may invade the surrounding tissue and spread, one could subsequently incorporate spatial effects by studying partial differential equations (PDEs) [5, 8]. Cancer cells grow exponentially in early stages due to sufficient supply of oxygen and nutrients [9–11]. Then growth decreases until the population size reaches its carrying capacity after nutrient supply is no longer enough, which is represented by the logistic [4, 12] and Gompertz models [13, 14]. These ODEs can be used to describe the interaction between cancer growth and therapy by adding an anticancer treatment term. With constant drug concentration, the exponential model predicts that cancer will continuously grow. However, the logistic and Gompertz models show that therapy will hold the cancer to some maximum size depending on the values of the parameters [7].

To eradicate cancer, oncologists use anticancer drugs, which either slow down or block the cell division cycle causing cell death [15]. These drugs are considered toxic because they attack rapidly growing cells including skin [16], gut [17], and bone marrow [18]. One anticancer treatment protocol includes a series of scheduled doses (conventional bolus treatment) administered intravenously into the blood stream [19]. Another protocol releases a drug at a constant rate through, for example, nanoparticles [20]. Mathematical modeling suggests that the effect of this constant delivery depends on the initial size of the cell population when the drug is first given [10]. Moreover, a continuous infusion is more effective than bolus applications because of the higher uptake rate [21] and because cancer cells proliferate between doses [22]. This kind of drug delivery exposes the healthy tissue to an extensive amount of toxicity without allowing them to regrow. This can be avoided by developing drugs targeting only cancer cells. Choosing the therapeutic strategy depends on the type of cancer. If the cancer has drug-resisting cells, then mathematical modeling indicates that a bolus dose is more effective as the cancer responds to it faster than a drug given continuously. The two regimes yield the same result for cancer with drug susceptible cells [8].

Most of the mathematical models describe the evolution of cancer as a spatially uniform mass, which grows at a fixed rate. In this paper, we consider the spatial influences on the dynamics between cancer and chemotherapy with constant drug delivery. Specifically, we develop the coupled PDE for drug-cell interaction and drug diffusion and perfusion [23] by considering an extra biological effect, which is cell proliferation. These equations represent a more realistic situation since highly vascularized cancers can proliferate between doses. Model predictions are given through numerical simulations for different values of the key biological parameters (proliferation rate, radius of the blood vessel, diffusion length of the drug, and blood volume fraction) along with the ratio of the viable cancer mass to its initial mass after giving the drug. These simulations represent cancer response for a continuous drug delivery but are not limited to this kind of drug method. Our results provide the opportunity to understand the interaction between cancer and chemotherapy. They can be used as a basis to model more complicated situations or as an alternative therapeutic strategy such as immunotherapy.

## 2. Methods

### 2.1. Mathematical Model

In our mathematical model, we add complexity to the PDEs representing the drug-cancer interaction (with the same assumptions) [23] by adding a proliferation term. We assume that the cancer is vascularized with enough nutrients and oxygen creating an ideal environment for cancer to grow at a rate proportional to its density (with and without treatment).

The first equation in the coupled PDEs represents diffusion of the drug into the cancer after it is delivered through the blood vessel and the binding rate to cancer cells. The second equation represents the death rate caused by the drug and the growth rate of cancer cells. The death rate is proportional to the history of drug uptake by cancer cells. After the cells uptake the drug, it will typically damage the DNA. Thus the increasing uptake over time causes more damage across the cell population and an increase in cell death [21, 23]. This represents the only death mechanism caused by the drug. We assume that the growth rate is a constant, although it may depend on the type of cancer or its density; therefore the tumor grows exponentially without treatment at a constant rate. We will study the model for a cylindrically symmetric domain with an infinite radius, where the cancer is initially homogenous and the drug has a constant concentration at the blood vessel with no flux at infinity.

The mathematical model is given by

(1) ∂σ∂t=D∇2σ−λuφσ,

(2) ∂φ∂t=−λuλkφx,t∫0tσx,τφx,τdτ+αφ,

where *σ*(**x**, *τ*) is the drug concentration, *φ*(**x**, *τ*) is the density of cancer cells, *D* is the drug diffusivity, *λ*_*u*_ is the cellular uptake rate of drug per-volume, *λ*_*k*_ is the death rate of tumor cells per unit cumulative drug concentration, and *α* is the growth rate of cancer cells. Since the diffusion rate of the drug is faster than the cell cycle, then the time derivative in (1) is replaced by zero (because it does not depend on time). Therefore, we need to find the quasi-steady state solution of (1) given by *φ*. Thus we need boundary conditions for (1) and an initial condition for (2).

We assume that the domain surrounding the blood vessel is cylindrically symmetric. This means that the system depends on two parameters: time and radial distance *r*. At the blood vessel, there is a constant rate of drug release *σ*_0_, for example, through nanocarriers. If *r* → *∞* there is no flux (the tumor is infinitely sized). Accordingly, we have the following initial and boundary conditions:

(3) φx,0=φ0,σrb,t=σ0,n·∇σx→∞⟶0,

where *r*_*b*_ is the radius of the blood vessel and *φ* is initially homogenous.

### 2.2. Nondimensionalizing

Before we numerically solve the model, we nondimensionalize the system to determine the key parameters. Thus, we get

(4) ∇′2σ′−φ′σ′=0,

(5) ∂φ′∂t′=−φ′x′,t′∫0t′σ′x′,τφ′x′,τdτ+α′φ′,

(6) φ′x′,0=1,

(7) σ′rbL,t′=1,

(8) n′·∇′σ′x′→∞⟶0,

where the dimensionless variables are **x**′ = **x**/*L*, *t*′ = *t*/*T*, *σ*′ = *σ*/*σ*_0_, *φ*′ = *φ*/*φ*_0_, *T* = (*λ*_*k*_*λ*_*u*_*φ*_0_*σ*_0_)^−1/2^, $L=\sqrt{D/\left(\varphi_{0}\lambda_{u}\right)}$, and *α*′ = *αT*. *T* is the time of the apoptotic cycles caused by the drug [21] and *L* is the diffusion length of the drug.

We assume that cancer cells depend on the closest blood vessel, which has dimensionless radius *r*_*b*_/*L*. Therefore, we estimate the dimensionless radius of the cylindrical region supported by the blood vessel by $r_{b}/\left(L\sqrt{BVF}\right)$ [6, 23]. BVF is the blood volume fraction (that is the ratio of the volume of blood to the volume of the tumor), which is less than 1. A higher value of BVF represents a highly vascularized tumor; this means that there are more blood vessels and therefore more treatment will be delivered to the tumor. Therefore, (8) can be rewritten as

(9) $$\begin{array}{c} {\left.\;\frac{d\sigma^{\prime }}{dr^{\prime }}\;\right|}_{r^{\prime }=r_{b}/\left(L\sqrt{BVF}\right)}=0. \end{array}$$

Note that we will drop the dash for simplicity.

### 2.3. Long-Term Response

After a long time of treatment, the cancer cells will be saturated with the drug and the death rate becomes a constant. Since *σ* is a finite continuous series of treatments, then by taking *t* → *∞*, the time integration of drug uptake is ∫_0_^*∞*^*σ*(**x**, *τ*)*φ*(**x**, *τ*)*dτ* = *μ*; and hence from (5) we get *φ* = *e*^(*α*−*μ*)*t*^. This means that the tumor will grow or decay exponentially depending on the values of *α* and *μ*. If *α* > *μ*, then cancer will continuously proliferate. On the other hand, if *α* < *μ*, then cell death overcomes cell growth. Otherwise, if *α* = *μ*, we have a quiescence state since cancer progression is balanced with cancer death.

### 2.4. Numerical Solution

We numerically simulate (4) and (5) with the initial and boundary conditions given by (6), (7), and (9). After nondimensionalizing, the only parameters in theses equations are *α*, *r*_*b*_/*L*, and BVF which are biological parameters. Small values of *r*_*b*_/*L* represent large diffusion of the drug if we fix *r*_*b*_; and large values of BVF represent tumor with high vascularization. First, we discretize *φ* and *σ* in space and then we solve (5) at each time step using fourth-order Runge-Kutta Method [24], where *σ* is given. The latter is calculated by solving (4) (using finite difference method [25]), where *φ* is known from the previous time step.

### 2.5. Calculating the Ratio of the Viable Cancer Mass to the Initial Mass

First, we integrate the density of the viable cancer cells at each time step over the cylindrically symmetric domain around the blood vessel (after drug diffusion). This is done during the numerical simulation (explained in the previous section). Then, we calculate the ratio of the viable cancer mass *M* to the initial mass *M*_0_ as follows:

(10) $$\begin{array}{c} f\left(\;t\;\right)=\frac{M}{M_{0}}=\frac{2\pi }{V_{0}}\overset{r_{b}/\left(L\sqrt{BVF}\right)}{\underset{r_{b}/L}{\int }}\varphi r\;dr. \end{array}$$

The initial mass is equal to the initial volume of the tumor, since *φ* = 1 at *t* = 0, which is given by $V_{0}=\pi \left[{\left(r_{b}/\left(L\sqrt{BVF}\right)\right)}^{2}-{\left(r_{b}/L\right)}^{2}\right]$.

## 3. Results

**Figure 1:**
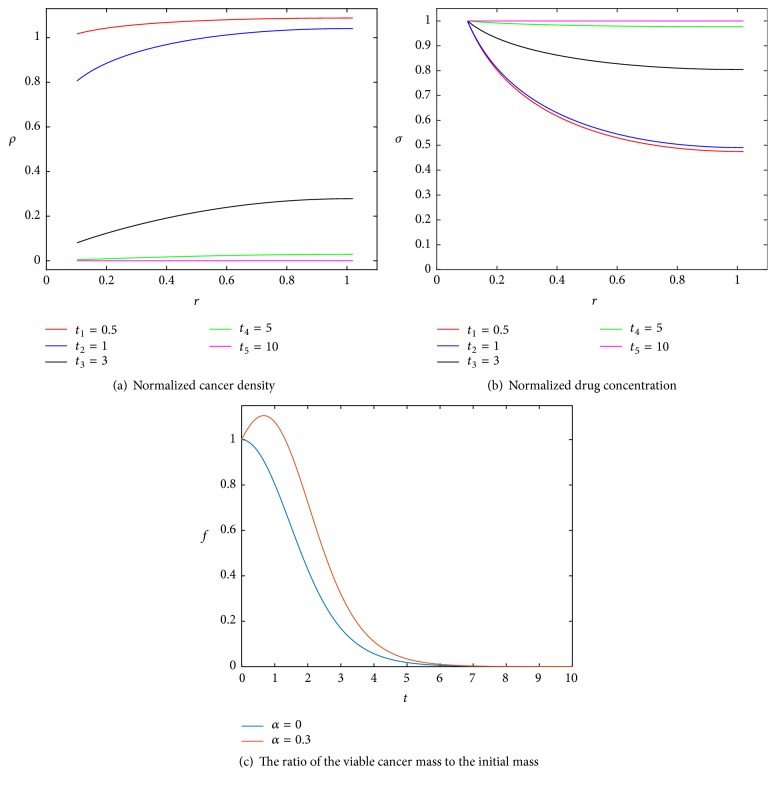
(a) Numerical simulations of (4)–(7) and (9), where BVF = 0.01, *r*_*b*_/*L* = 0.102, and *α* = 0.3. Here *r* and *t* are the dimensionless radial distance and time, respectively. In (c), *f* is plotted against *t* for *α* = 0 and *α* = 0.3. For the latter, *f* increases at the beginning of the treatment due to proliferation; then after a short time the drug overcomes proliferation and cancer cells all die after 6 apoptotic cycles.

We numerically solve (4)–(7) and (9), for BVF = 0.01, *r*_*b*_/*L* = 0.102 (same as in [23] to compare the results), and *α* = 0.3 for 10 apoptotic cycles (caused by the drug). Note that with *α* = 0 we get the same model as in [23]. The solution in Figure 1(a) shows that, at the beginning of the simulation, cancer cells near the blood vessel wall die (due to drug penetration) and further away cells proliferate. Then, we get a similar result as in the case for *α* = 0, where the killing of cancer cells increases causing also an increase in the drug concentration (Figure 1(b)) killing more cells. In Figure 1(c) (for *α* = 0.3), the ratio of the viable cancer mass to the initial mass increases at the beginning of the treatment due to proliferation; then after a short time, the drug overcomes proliferation and all cancer cells die after 6 apoptotic cycles, which is similar to the number of cycles needed for *α* = 0.

**Figure 2:**
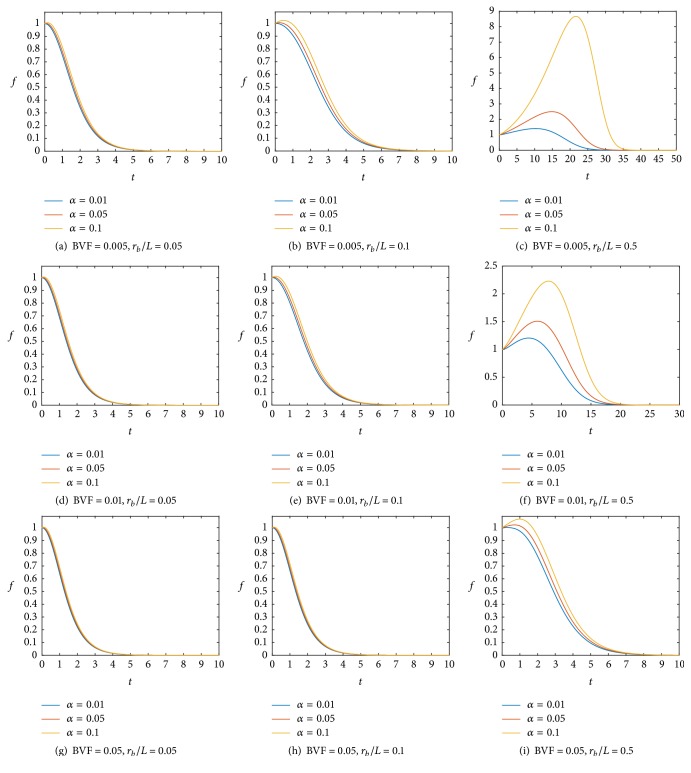
Temporal evolution curves of the ratio of the viable cancer mass to the initial mass calculated numerically from (10) with different values of BVF and *r*_*b*_/*L* as shown under each figure. The values of *α* are given in the legend of each graph.

Now we vary the parameters BVF, *r*_*b*_/*L*, and *α* and numerically calculate the value of the ratio of the viable cancer mass to the initial mass as shown in Figure 2. Note that the values of *α* are indicated in the legend of each graph and the values of BVF and *r*_*b*_/*L* are given under each figure (values same as in [23]). In each figure, the values of BVF and *r*_*b*_/*L* are fixed and the value of *α* is varied. As the value of *α* is increased in each figure more cells will proliferate, and cancer cells will continue growing. However, at some point, the continuous release of the drug will cause the cells to stop proliferating and then all cells will die. If we compare the figures from left to right (*r*_*b*_/*L* increases which means less diffusion of the drug if we fix *r*_*b*_ and BVF is fixed), we find that cancer progresses more and the drug needs to be given for a longer period of time. Moreover, there is a noticeable difference between different values of *α* (in each figure) on the growth of cancer. For example, in Figure 2(a), all values of *α* almost have the same effect on the growth and cancer cells die after a short time. However, in Figure 2(c) there is a distinct result for each case and the drug becomes successful after a long time. If we compare the figures from top to bottom (i.e., BVF increases which represents highly vascularized cancer and *r*_*b*_/*L* is fixed), we get a better result in which cancer is killed in a shorter period of time. Moreover, proliferation becomes closer for the different values of *α* in each figure. Therefore, as suggested by [23], increasing angiogenesis or perfusion by, for example, hyperthermia, will improve the result of treatment.

## 4. Discussion (Implementation and Future Work)

We have added a proliferation term to the PDE representing the interaction between cancer density and drug concentration. Then we performed numerical simulations for different values of the parameters: proliferation rate, radius of the blood vessel, diffusion length of the drug, and blood volume fraction. We found that a continuously administered drug is more effective if the tumor is highly vascularized (which means more exposure to the treatment) or if the penetration length of the drug is high. In this case, the drug overcomes proliferation and the cancer is killed in a short time. This result suggests increasing angiogenesis or perfusion. This is similar to the case where proliferation is neglected because the continuous application of the drug outweighs the effect of cancer growth.

**Figure 3:**
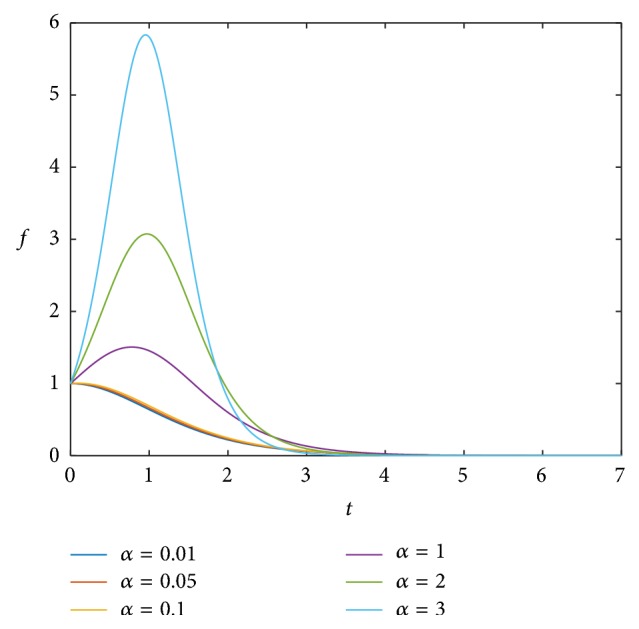
Temporal evolution curves of the ratio of the viable cancer mass to the initial mass calculated numerically from (10) with different values of *α* as given in the legend, where BVF = 0.05 and *r*_*b*_/*L* = 0.05.

From our result, it seems that when BVF is high and *r*_*b*_/*L* is low, the treatment is successful even if we increase the value of *α* as shown in Figure 3; however, we need to know the extent to which we can increase this value (also, for different values of BVF and *r*_*b*_/*L*). This means finding the threshold value of *α*, such that above this value the drug is no longer effective. In Figure 3, we chose the highest value of BVF and the lowest value of *r*_*b*_/*L* from Figure 2 and increased the value of *α*. As the growth rate increases, the ratio of the viable cancer mass to the initial mass also increases at the beginning of the simulation. Then after approximately the same number of apoptotic cycles, cancer cells die for all chosen values of *α*. It is useful to estimate the values of the parameters from in vivo or in vitro experiments for different kinds of cancer and validate the model with patient's data so that it can be used to predict the outcome of the treatment. This will guide oncologists to choose the optimal therapy with minimal patient suffering.

Future work could also include adding physiological or biological complexity to the coupled PDEs. For example, instead of choosing the proliferation rate as a constant, it could depend on the size of the tumor [4, 26, 27]; thus the growth term can be represented by the logistic or Gompertz growth. Our model was studied with a continuous delivery of the drug from the blood vessel. We can also investigate the situation where the drug is given as a bolus dose in repeated cycles then compare the two results. If the proliferation of cells is neglected, then experimental data and the mathematical model show that there is a 3-fold increase in response for the continuous delivery of the drug compared to the bolus treatment [23]. Thus, if cell growth is taken into account, it is expected to get a better response for the continuous infusion of the drug. This is because cancer cells might proliferate between the doses of the bolus treatment and the continuous delivery of the drug will overcome the proliferation.
